# Genetic Diversity and Epidemic Types of Porcine Reproductive and Respiratory Syndrome (PRRS) Virus in Japan from 2018 to 2020

**DOI:** 10.3390/epidemiologia3020022

**Published:** 2022-06-03

**Authors:** Fumiaki Kyutoku, Takashi Yokoyama, Katsuaki Sugiura

**Affiliations:** 1 Environmental Science for Sustainable Development, Graduate School of Agricultural and Life Sciences, The University of Tokyo, 1-1-1 Yayoi, Bunkyo-ku, Tokyo 113-8657, Japan; fumiaki.kyutoku@boehringer-ingelheim.com (F.K.); ayokoyam@g.ecc.u-tokyo.ac.jp (T.Y.); 2 Boehringer Ingelheim Animal Health Japan Co., Ltd., 2-1-1 Osaki, Shinagawa-ku, Tokyo 141-6017, Japan; 3 Nippon Institute for Biological Sciences, 9-222-1 Shin-machi, Ome, Tokyo 198-0024, Japan

**Keywords:** genetic diversity, phylogenetic analysis, porcine reproductive and respiratory syndrome, sequence identity

## Abstract

To clarify the genetic diversity of the porcine reproductive and respiratory syndrome virus (PRRSV) in Japan in recent years, we determined the nucleotide sequence of open reading frame 5 of 2482 PRRSV sequences obtained from samples collected from pigs between January 2018 and December 2020. As a result of molecular phylogenetic analysis, Cluster II represented the largest proportion (44.9–50.6%) throughout the study period, followed by Cluster IV (34.0–40.8%), Cluster III (7.8–12.1%), Cluster I (3.1–6.7%), and Cluster V (0.1–0.2%). The relative distributions between Clusters varied between geographic regions and between years: in 2018, Cluster II was the most prevalent in all regions. In 2019, Cluster II was dominant in the Hokkaido and Tohoku regions, while in other regions Cluster IV was dominant. In 2020, Cluster IV was dominant in the Kanto/Tosan and Kyushu/Okinawa regions, whilst in other regions Cluster II was predominant. Compared with a previous study, the proportions of genome sequences classified in Clusters II and IV significantly increased (*p* = 0.042 and 0.018, respectively) and those classified in Cluster III significantly decreased (*p* < 0.01). The widespread use of live attenuated vaccines using strains that belong to Cluster II might have accounted for these changes in the relative distribution between Clusters.

## 1. Introduction

The porcine reproductive and respiratory syndrome virus (PRRSV) causes porcine reproductive and respiratory syndrome (PRRS), which is characterized by reproductive failure in sows and respiratory disease in piglets [1]. PRRSV is a member of the family *Arteriviridae*, order *Nidovirales* [2]. PRRSV is a spherical enveloped virus 50–72 nm in diameter with a nucleic acid of about 15.1–15.4 kb with single-stranded RNA [3]. PRRS was first reported in North America in 1987 [4] and subsequently in European countries: Germany and the Netherlands in 1990; Belgium, Britain, and Spain in 1991; and later in other European countries [5]. PRRS has spread across the globe. The PRRSV was first detected in 1991 in the Netherlands [6], and thereafter in the United States [7]. There are two major genotypes of PRRSV: the North American type (or Type II, prototype strain VR2332) and the European type (or Type I, prototype strain Lelystad) [8,9,10,11,12]. The Type II virus has spread widely since the disease was first reported in Japan in 1994 [11].

It has also been suggested that PRRSV is associated with and is one of the key pathogens of the Porcine Respiratory Disease Complex (PRDC) [13,14,15], a complex infection with other respiratory infections, such as the porcine circovirus and porcine mycoplasma infections. The economic losses caused by PRRS in Japan were estimated to be JY 25.46 billion in 2018 [16].

The diagnosis of PRRS depends on the measurements of enzyme-linked immune-sorbent assay (ELISA) antibody titers and the detection of the PRRSV antibody by a immunoperoxidase monolayer assay or an immunofluorescence assay of blood or serum samples collected from pigs at different production stages; detection of PRRSV genes by reverse transcription-polymerase chain reaction test (RT-PCR); the detection of antigens in the lungs and tonsils by immunohistochemical staining; and the isolation of PRRSV using porcine alveolar macrophages [17,18,19,20,21]. Among these, gene detection by RT-PCR is an important tool for the early diagnosis and monitoring of PRRSV infection on farms where vertical and horizontal transmission is suspected. In addition to blood and serum samples, oral fluid and processing fluid samples obtained from newborn male pigs at the time of castration are used for gene detection by RT-PCR [22,23].

PRRSV contains at least nine open reading frames (ORFs) including ORFs 1a and 1b, ORFs 2a and 2b, and ORFs 3–7 [24]. Among these, ORF7 encodes the N protein, which is conserved among strains and is routinely used for target diagnosis [25,26]. ORF5 encodes the viral surface glycoprotein GP5, a commonly recognized antigen in animals exhibiting protection against PRRSV and an excellent candidate protein for producing a recombinant vaccine [27]. This region is particularly prone to mutation and is used as an indicator for the genotyping of PRRSV worldwide [23,24,25,26,27,28,29,30,31]. An increased phylogenetic variation was revealed previously using the ORF5 gene analysis of strains detected in Japan between 2007 and 2008 [32].

This study aimed to identify the diversity of Type II PRRSV genes and epidemic types distributed in Japan in recent years by determining the nucleotide sequences of the ORF5 genes using sequences from samples collected in Japan between January 2018 and December 2020.

## 2. Materials and Methods

### 2.1. Gene Extraction and Sequencing

A total of 2482 PRRSV nucleotide sequences of the ORF5 genes detected from samples collected from pigs during the years from 2018 to 2020 were used for molecular phylogenetic analysis based on the method described in a previous study [32]. The number of farms from which these sequences were detected, and their geographic distributions are shown in Table 1. In the majority of these farms, sows and gilts were vaccinated twice during the gestation using modified live vaccines (MLV). An average of 3.1–3.2 samples were collected from each farm (Table 1). These PRRSV nucleotide sequences were detected mostly from blood or serum specimens and partly from oral fluid, processing fluid, and sperm specimens collected from live pigs, and were used to investigate the transmission and circulation of PRRSV within farms (Table 2). No information was available on whether these pigs exhibited clinical signs at the time of collection of samples. In addition, there were genome sequences detected from specimens obtained from organs such as lung/hilar lymph nodes, tonsils, lymph nodes, aborted fetus, and pleural effusion that were collected for pathological evaluation by necropsy (Table 2). These samples were collected and analyzed as a part of the diagnostic service provided by Boehringer Ingelheim Animal Health Japan Co., Ltd. (Tokyo, Japan) to its client farmers. The samples were subjected to ribonucleic acid (RNA) extraction and amplification in the ORF7 gene region by RT-PCR using the method previously described [25,26] at Shokukanken Inc. (Maebashi City, Japan), a private testing laboratory. RT-PCR positive samples were further amplified by real-time PCR in the ORF5 gene region using the primer sets described previously [25,26,28].

### 2.2. Phylogenetic Tree Construction and Cluster Classification

Molecular Evolutionary Genetic Analysis (MEGA) software version 7.0 (Institute for Genomics and Evolutionary Medicine, Temple University, PA, USA) was used to create a phylogenetic tree for each PRRSV sequence for which the ORF5 gene region was sequenced [33]. Of the 2482 sequences, 2425 sequences had an ORF5 gene sequence length of 603 bp and 57 sequences had an ORF5 gene sequence length of 600 bp. Therefore, the length of the ORF5 gene sequence was adjusted to 588 bp by removing the regions from 94 bp to 108 bp for sequences with a length of 603 bp, and the regions from 94 bp to 105 bp for sequences with a length of 600 bp, using the method described by Iseki et al. [32]. The molecular phylogenetic tree was constructed by determining the evolutionary distance using the Tamura–Nei model after 1000 replications of bootstrap method analysis and using the neighbor joining method based on the arranged sequences. We then classified each specimen into Clusters I to V as in the previous study [32]. Phylogenetic analyses of the sequences detected in this study were conducted using genomic information of 30 Japanese strains previously obtained and 11 North American type viruses detected from around the world. In addition, we investigated the relative distribution between Clusters by geographic region over the years between 2018 and 2020.

### 2.3. Identity of PRRSV Genome Sequences Detected in This Study with Reference Strains

As in the previous study [32], PrimePac^®^ PRRS strain (Merck Animal Health, Madison, NJ, USA), Ingelvac^®^ PRRS MLV strain (Boehringer Ingelheim Vetmedica Inc., Duluth, GA, USA), EDRD-1 strain (National Institute of Animal Health, Tsukuba, Japan), Jpn5-37 strain (National Institute of Animal Health, Tsukuba, Japan), and Jos1 strain (National Institute of Animal Health, Tsukuba, Japan) were used as the reference strains for Clusters I, II, III, IV and V, respectively. For Cluster I, Fostera^®^ PRRS strain (Zoetis Animal Health US, Parsipanny, NJ, USA), which has been available in Japan since 2018, was also used as a reference strain. EDRD-1, Jpn5-37 and Jos1 were the first wild type isolates detected for each Cluster in Japan and commonly used as reference for phylogenetic analysis (Table 3) [32,34]. A molecular biological analysis software, BioEdit (University of Bahri, Khartoum, Sudan) [35], was used to examine the homology between the sequences classified in each Cluster and the reference strain. The identity with the reference strain within each Cluster was then compared over the three study years.

### 2.4. Statistical Analysis

All statistical analyses were performed with EZR version 1.40 (Saitama Medical Center, Jichi Medical University, Saitama, Japan) [36], a graphical user interface for R version 3.6.0 (The R Foundation for Statistical Computing, Vienna, Austria).

A chi-square test was used to investigate if there was a significant difference between the relative distributions of PRRSV sequences classified in the Clusters in this study and previous studies, and between Clusters in the respective geographic regions. When comparing the identity distributions of sequences classified in each Cluster between years, the Shapiro–Wilk test was first used to check the normality of the identity distribution within Clusters. If the sequences within Clusters were normally distributed in terms of identity, one-way analysis of variance (ANOVA) was performed to draw a comparison between the years. If normality was not assumed for any of the three years, the Kruskal–Wallis test was used for between-year comparisons, followed by Steel–Dwass multiple comparisons in post-hoc tests. All *p*-values less than 0.05 were considered statistically significant.

## 3. Results

### 3.1. Predominant Type and Cluster

**Figure 1:**
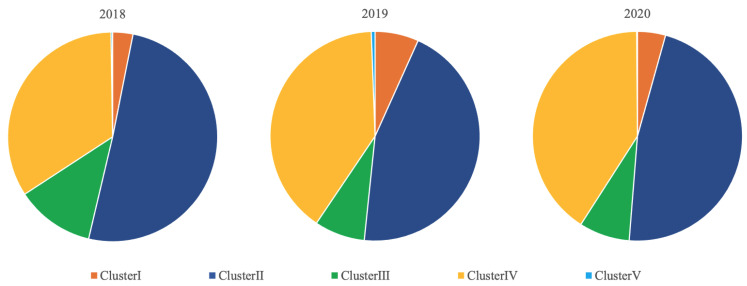
Relative distribution of PRRSV ORF5 genes detected from 2018 to 2020 and analyzed in this study between different Clusters.

All the PRRSV sequences detected in this study were of the North American type, also called PRRSV Type II. The relative distribution between Clusters for the three study years at the national level are shown in Figure 1. Throughout the study years, Cluster II represented the highest proportion (44.9–50.6%), followed by Cluster IV (34.0–40.8%), Cluster III (7.8–12.1%), Cluster I (3.1–6.7%), and Cluster V (0.1–0.2%).

### 3.2. Distribution of Predominant Cluster by Region

**Figure 2:**
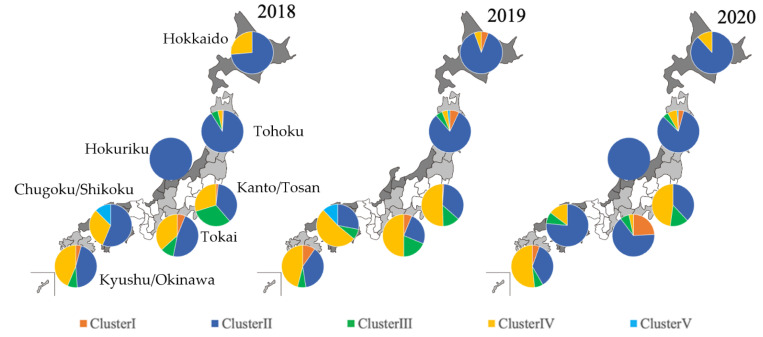
Relative distribution of PRRSV ORF5 genes detected from 2018 to 2020 and analyzed in this study by admirative region between different Clusters. Each patterned circle represents a region of Japan.

The relative distributions between the Clusters for the seven geographic regions (Hokkaido, Tohoku, Kanto/Tosan, Hokuriku, Tokai, Chugoku/Shikoku, and Kyushu/Okinawa) for each of the three years 2018, 2019, and 2020, are shown in Figure 2. In 2018, Cluster II was dominant in all regions; followed by Cluster III in the Tohoku and Kanto/Tosan regions; then Cluster IV in the Hokkaido, Tokai, Chugoku/Shikoku, and Kyushu/Okinawa regions. In 2019, Cluster II was dominant followed by Cluster IV in the Hokkaido and Tohoku regions. In other regions, Cluster IV was dominant, followed by Cluster I. In 2020, Cluster II was dominant, followed by Cluster IV in the Hokkaido, Tohoku, Hokuriku, and Chugoku/Shikoku regions. In the Tokai region, Cluster II was dominant, followed by Cluster I. In the Kanto/Tosan and Kyushu regions, Cluster IV was dominant, followed by Cluster II. All these dominances were statistically confirmed to be significant.

### 3.3. Genetic Diversity of Each Cluster to the Reference Strain

**Figure 3:**
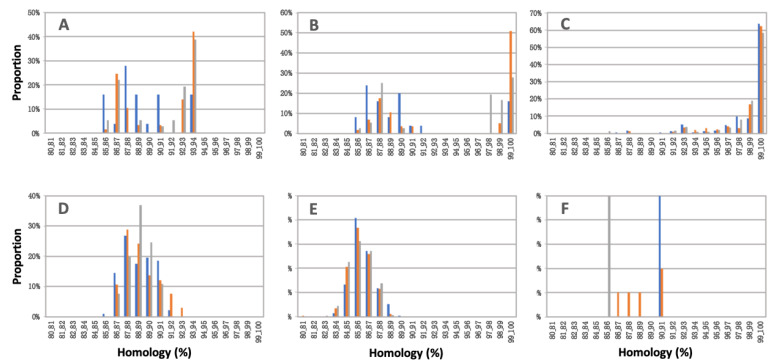
Homology distribution of PRRSV isolates classified in Cluster I (**A**,**B**), Cluster II (**C**), Cluster III (**D**), Cluster IV (**E**), and Cluster V (**F**) detected from 2018 to 2020. The reference strains used were Prime Pac^®^ MLV (**A**) and Fostera^®^ PRRS (**B**) for Cluster I, Ingelvac^®^ PRRS MLV for Cluster II, EDRD-1 for Cluster III, Jpn5-37 for Cluster IV, and Jos1 for Cluster V. Blue, orange, and gray bars indicate 2018, 2019, and 2020, respectively.

Figure 3 and Table 4 show the identity distributions of the PRRS sequences classified in Clusters I, II, III, IV, and V, calculated for each of the three study years using Prime Pac^®^ PRRS MLV and Fostera^®^ PRRS, Ingelvac^®^ PRRS MLV, EDRD-1, and Jpn5-37 and Jos1 strain as reference strains, respectively.

The sequences classified in Cluster I showed a bimodal distribution with a low nucleotide identity (85–94%) to the Prime Pac^®^ PRRS MLV strain (Figure 3A). They also showed a bimodal distribution with 85–100% nucleotide identity values when the Fostera^®^ PRRS strain was used as a reference (Figure 3B), with one peak with high nucleotide identity values (97–100%) for the reference strain.

The sequences classified in Cluster II showed a unimodal distribution with high identity values with the Ingelvac^®^ PRRS MLV strain throughout the study years (Figure 3C).

The sequences classified in Clusters III and IV showed a normal distribution, with 85–93% and 83–89% nucleotide identity values for the reference strains, respectively (Figure 3D, E).

The sequences classified in Cluster V were not significantly numerous enough to show any distribution pattern (Figure 3F).

The sequences classified in Clusters I and IV showed a significant difference between study years in their identity distributions compared to the respective reference strains: a significant increase in identity to the reference strain was observed between 2018 and 2019 for Clusters I and IV (Table 4).

## 4. Discussion

In this study, we conducted an epidemiological survey based on a molecular phylogenetic analysis on the ORF5 sequence of 2482 PRRSV sequences detected in 32 prefectures in Japan during the three-year period from 2018 to 2020, with the aim of clarifying the genetic diversity of PRRSV originating on pig farms in Japan. The PRRSV sequences analyzed in this study were not collected from samples randomly selected from pig farms in Japan but detected from convenience samples collected under a diagnostic program provided by a private vaccine manufacturer. Therefore, our samples might not be representative of the general pig population in Japan. In addition, considering that the number of farms analyzed in this study only represented 5.8–5.9% of the total pig farms in Japan, the findings of this study cannot be generalized to the pig population in Japan. However, they provide valuable insights into the diversity and the epidemic type of PRRSV in Japanese pig herds.

Our study revealed that the relative distributions between the Clusters of PRRSV were relatively stable during the three years, with Clusters II and IV representing the largest proportions, followed by Clusters III, I, and V. A previous study reported that Cluster III represented the highest proportion based on the analysis of PRRSV strains detected in 2007 and 2008 [37]. Although not totally comparable because our sequences were derived from samples collected from both healthy and necropsied pigs, while their isolates were detected from diseased pigs, our results indicated a significant increase in the proportion of Clusters II and IV (*p* = 0.042 and 0.018, respectively) and a significant decrease in the proportion of Cluster III (*p* < 0.01) (data shown in Table S1). Given that the PRRSV sequences classified in Cluster II in this study are genetically very close to the Ingelvac^®^ PRRS MLV strain, the increased circulation of Cluster II strains might have been caused by the widespread use of this vaccine for routine vaccination programs. To the contrary, the PRRSV sequences classified in Cluster IV were not closely homologous to the reference strain (Jpn5-37) with some sequences presenting an identity of up to 90.6%. Although the Jpn5-37 strain has been experimentally proven to be highly virulent [37], Cluster IV strains now circulating in Japan might not be virulent but highly infectious, thus resulting in viruses of this Cluster spreading in the pig population without killing the host animals.

Interestingly, the identity distributions to the reference strain of the sequences classified as Clusters I and II might be consistent with known vaccination practices in Japan. The Fostera^®^ PRRS strain, one of the reference strains for Cluster I in our study, is a vaccine strain approved and marketed in Japan since 2018 and it has been used by pig farmers for PRRS control. This might have resulted in the detection of many viruses closely related to Fostera^®^ PRRS during the study period. To the contrary, there were no Cluster I sequences detected in this study that were closely related to Prime Pac^®^ PRRS MLV, another reference strain. This is most likely because Prime Pac^®^ PRRS MLV has not been approved and used commercially in Japan.

Regarding Cluster II, the majority of the sequences classified in this Cluster revealed over 95% sequence identity with the Ingelvac^®^ PRRS MLV strain. This is most probably because this vaccine has been approved in Japan for more than 20 years and is still widely used by many pig farmers to protect their pig herds from the field strains of PRRSV that cause clinical diseases. The widespread use of this vaccine might have led to the increase in Cluster II strains. Currently, the only modified live PRRS vaccines used on pig farms in Japan are Fostera^®^ PRRS, produced using a Cluster I strain, and Ingelvac^®^ PRRS MLV, produced using a Cluster II strain. As shown in a previous study in the United States [38], the continued use of these vaccines might lead to the increase of strains that are highly identical with the respective vaccine strains.

No significant change was observed in the identity distribution of the Cluster III sequences over the study period, suggesting that no significant genetic change from the reference strain occurred during these three years. The EDRD-1 strain used as the reference strain for Cluster III in this study was detected in Japan in 1993. The long period that has elapsed since then might have lowered the identity of this Cluster’s sequences compared with Clusters I and II’s sequences, for which the vaccine strain was used as the reference strain. The identity of the sequences classified in Cluster IV with the reference strain was low, suggesting that the strains have been propagated and mutated repeatedly from the time they were first detected in Japan to the present. Two sequences and one sequence classified in Cluster IV in this study were close to a strain detected in Austria (AY875854) and a strain detected in Denmark, respectively, (AF095515), each with an identity being above 99%. However, no epidemiological evidence was available that could possibly link these sequences with pigs imported from either of these countries. The number of Cluster V sequences detected in this study was too small to draw inference using statistical analysis.

In this study, we used MEGA to create a phylogenetic tree for each of the PRRSV strains for which the ORF5 gene region was sequenced. However, there are other methods used to describe virus diversity and evolution such as the lineage system using a Bayesian Markov chain Monte Carlo (BMCMC) method [39,40]. Further studies are needed to describe virus diversity and evolution using this method.

## 5. Conclusions

Our study indicated that PRRSVs on pig farms in Japan have a high degree of genetic diversity and that this diversity has increased over time in the past decade. In addition, our study revealed that the epidemic type of PRRSV has changed over the past decade. A recent study conducted in Canada using whole-genome sequencing was more successful than ORF5 sequencing, in obtaining strain classification and interpretation of results in 9.10% of clinical samples [41]. The whole-genome sequencing of the 2,482 samples analyzed in the current study might result in a better classification of PRRSV which could lead to more appropriate interventions by veterinarians and pig farmers. Nevertheless, it is important to monitor the genetic diversity of PRRSV to elucidate whether there is a temporal change in the PRRSV genes in order to obtain further information for the effective control of the disease.

## Figures and Tables

**Table 1:** The numbers of porcine reproductive and respiratory syndrome virus (PRRSV) ORF5 genes detected from 2018 to 2020 and analyzed in this study by region and number of prefectures and farms.

Administrative Region	2018			2019			2020		
	Prefectures	Farms	Detects *	Prefectures	Farms	Detects *	Prefectures	Farms	Detects *
Hokkaido	1	6	19	1	9	37	1	8	34
Tohoku	5	30	112	6	48	114	6	38	95
Kanto/Tosan	6	53	168	5	49	200	7	52	184
Hokuriku	1	1	2	0	0	0	1	2	3
Tokai	4	21	49	3	9	16	2	12	29
Kinki	0	0	0	0	0	0	0	0	0
Chugoku/Shikoku	3	6	16	3	7	25	4	7	34
Kyusyu/Okinawa	8	138	435	8	146	456	7	139	454
Total	28	255	801	26	268	848	28	258	833

* Number of samples from which PRRSV ORF5 genes were detected.

**Table 2:** The origin of porcine reproductive and respiratory syndrome virus (PRRSV) ORF5 genes detected from 2018 to 2020 and analyzed in this study.

Origin	Specimen	2018	2019	2020
Live pig	Blood/serum	600	694	644
	Oral fluid	166	125	148
	Processing fluid	8	18	15
	Sperm	1	0	0
	Sub-total	775	837	807
Dead pig	Lung/Hilar lymph node	23	9	20
	Tonsil	1	0	0
	Lymph node	1	0	4
	Aborted fetus	1	2	1
	Pleural effusion	0	0	1
	Sub-total	26	11	26
Total		801	848	833

**Table 3:** Reference strains and sequences used in this study.

Name of Reference Strain	Cluster Type	Accession Number	ORF5 Gene Sequence
PrimePac^®^ PRRS	I	AF066384	atgttggggaaatgcttgaccgcgggttgttgctcgcgattgctttctttttggtgtatcgtgccgttctgttttgctgtgctcgtcaacgccagctacagcagcagctctcatttacagttgatttataacttgacgctatgtgagctgaatggtacagattggctggctaataaatttgattgggcagtggagagttttgtcatctttcctgtgttgacccacatcgtttcctatggtgcactaaccaccagccacttccttgacacagttggtctggttactgtgtctaccgccgggtttgttcatgggcggtatgtcctgagtagcatctacgcggtctgtgccctggctgcgttaatttgcttcgtcattaggttggcgaagaactgtatgtcctggcgctactcatgcaccagatacaccaactttcttctggacactaagggcagactctatcgttggcggtcgcctgtcatcatagagaaagggggtaaggtagaggtcgaaggccatctgatcgacctcaaaagagttgtgcttgatggttccgcggcaacccctttaaccagagtttcagcggaacaatggggtcgtccctag
Fostera^®^ PRRS	I	MK820650	atgttggggaaatgcttgaccgcgggctgttgctcgcgattgctttctttgtggtgtatcgtgccgttctggtttgctgtgctcggcaacgccaacagcagcagcagctctcatttccagttgatttataacttgacgctatgtgagctgaatggcacagattggctggcagaaaaatttgattgggcggtggagacttttgtcatctttcccgtgttgactcacattgtttcctattgtgcactcaccaccagccatttccttgacacagttggtctggttactgtgtccaccgccgggttttatcacgggcggtatgtcttgagtagcatctacgcggtctgtgctctggctgcgttgatttgcttcgttattaggcttgcgaagaactgcatgtcctggcgctactcttgtaccagatataccaacttccttctggacactaagggcagactctatcgttggcggtcgcccgttatcatagaaaaaaggggtaaggttgaggtcgaaggtcatctgatcgacctcaaaagagttgtgcttgatggttccgtggcaacccctttaaccagagtttcagcggaacaatggggtcgtctctag
Ingelvac^®^ PRRS MLV	II	AF020048	atgttggagaaatgcttgaccgcgggctgttgctcgcaattgctttctttgtggtgtatcgtgccgttctgttttgctgtgctcgccaacgccagcaacgacagcagctcccatctacagctgatttacaacttgacgctatgtgagctgaatggcacagattggctagctaacaaatttgattgggcagtggagagttttgtcatctttcccgttttgactcacattgtctcctatggtgccctcactaccagccatttccttgacacagtcgctttagtcactgtgtctaccgccgggtttgttcacgggcggtatgtcctaagtagcatctacgcggtctgtgccctggctgcgttgacttgcttcgtcattaggtttgcaaagaattgcatgtcctggcgctacgcgtgtaccagatataccaactttcttctggacactaagggcggactctatcgttggcggtcgcctgtcatcatagagaaaaggggcaaagttgaggtcgaaggtcatctgatcgacctcaaaagagttgtgcttgatggttccgtggcaacccctataaccagagtttcagcggaacaatggggtcgtccttag
EDRD-1	III	AB288356	atgttggggaaatgcttgaccgcgggctgttgctcgcgattgccttttttgtggtgtatcgtgccgttctgtcttgctgtgctcgtcaacgccagcgacagcagcagctcccatttacagttgatttataacctgacgctatgtgagctgaatggcacagattggctggctgacaaatttgattgggcagtggagagttttgtcatctttcccgtgttgactcacattgtttcttactgcgccctcactaccagccacttccttgacacagttggtctggtcgctgtgtctaccgccgggttttaccacgggcggtatgttctgagtagcatctatgcggtctgtgccctggctgcgttggtttgcttcgtcatcagattgacgaagaattgcatgtcctggcgctactcatgtaccagatataccaactttcttctggataccaagggcagactctatcgttggcggtcgcccgtcatcatagagaaagggggtaaggttgaggttgaaggtcatctgatcgacctcaagagagttgtgcttgatggttccgcggcaacccctataaccaaagtttcagcggaacaatggggtcatccctag
Jpn5-37	IV	AB546125	atgttggggaaatgcttgaccgcgggctattgctcgcaattgccttttttgtggtgtatcgtgccgttctgtcttgctgcgctcgtcaacgccaacagcaacagcagctcccatttacagttgatttataacttaacgatatgtgagctgaatggcacagattggctgaacaatcattttagttgggcagtggagactttcgttatctttcctgcgttgactcatattgtttcctacggcgcccttactaccagccacttccttgacacggtcggcctaatcactgtgtccaccgctggatactaccatgggcggtatgtcttgagtagcatctatgctgtctgcgccctggctgcgctgacttgcttcgtcatcaggttgacgaaaaattgtatgtcttggcgctactcatgtaccagatataccaactttcttctggacaccaagggcagactctatcgctggcggtcacccgtcattatagagaaaatgggtaaaattgaggttggaggtgacctgatcgacctcaagagagttgtgcttgatggttccgcggcaacccctgtaaccaaagtttcagcggaacaatggagtcgtccttag
Jos1	V	AB175699	atgttggggaaatgcttgaccgcgggctgttgctcgcgattgctttttttgtggtgtatcgtgccgtcctgttttgttgcgctcgtcagcgccaacaacagcagcagctctcatttacagttgatctacaacctgacgctatgtgagctgaatggcacagattggctggctaataaattcaattgggcagtggaaagttttgtcatctttcctgtgttgactcacattgtctcttatggtgcccttactaccagccatttccttgacacagtctgcttggtcactgtatctaccgccggtttttaccacgggcggtatgttttgagcagcatctacgcggtttgtgccctggccgcgttggtttgcttcgccgttaggttcgcgaagaattgcatgtcctggcgctattcgtgtaccaggtataccaactttcttctggacactaagggcagactctatcgttggcggtcgcctgtcatcatagagaaaaggggtaaagttgaggtcgaaggtcatctgatcgacctcaagagagttgtgcttgatggttccgcggcaacccctataaccaaaatttcagcggaacaatggggtcatctctag

**Table 4:** Homology distribution for referenced strain of PRRSV ORF5 genes detected from 2018 to 2020 and analyzed in this study by Cluster and year.

Cluster	Reference Strain	Year	Number of Isolates	Homology Distribution (%)	Mean (%)	SD *	Median (%)
Cluster I	PrimePac^®^	2018	25	85.3–93.3	88.8	2.5	88.0
		2019	57	85.0–93.5	90.4	3.1	92.6
		2020	36	85.3–93.7	90.7	3.0	92.2
Cluster I	Fostera^®^ PRRS	2018	25	85.7–99.8	89.9 ^a,^**	4.6	88.4
		2019	57	86.0–100.0	94.3 ^b,^**	5.8	99.1
		2020	36	85.2–99.8	94.6	5.6	97.8
Cluster II	Ingelvac^®^ PRRS MLV	2018	405	85.0–100.0	98.2	2.7	99.3
		2019	381	87.2–100.0	98.3	2.3	99.3
		2020	391	85.0–100.0	98.3	2.5	99.3
Cluster III	EDRD-1	2018	97	86.0–91.3	88.6	1.3	88.4
		2019	66	86.3–92.3	88.8	1.5	88.6
		2020	65	86.3–90.6	88.7	0.1	88.7
Cluster IV	Jpn5-37	2018	272	83.6–89.2	86.1 ^a,^**	1.1	85.8
		2019	339	80.4–88.9	85.8 ^b,^**	1.1	85.8
		2020	340	82.9–88.4	85.8	1.1	85.8
Cluster V	Jos1	2018	2	90.1–90.4	90.3	0.2	90.3
		2019	5	86.5–90.6	88.8	1.8	88.6
		2020	1	87.7	87.7	NT	87.7

Kruskal–Wallis test was used to investigate if there was a difference in the homology distribution between the years, followed by Steel-Dwass multiple comparisons in post-hoc tests. * standard deviation. ** Values with different letters (a, b) are significantly different (*p* < 0.05).

## Data Availability

The data presented in this study are available on request from the corresponding author.

## Supplementary Materials

The following supporting information can be downloaded at: https://www.mdpi.com/article/10.3390/epidemiologia3020022/s1; Table S1: The numbers of PRRSV genome sequences isolates analyzed in a previous study (Iseki et al.) and in this study.

## Abbreviations

PRRS	porcine reproductive and respiratory syndrome virus
PRRSV	porcine reproductive and respiratory syndrome virus
PRDC	porcine respiratory disease complex
ELISA	enzyme-linked immune-sorbent assay
RT-PCR	reverse transcription-polymerase chain reaction test
ORFs	open reading frames
RNA	ribonucleic acid
MLV	modified live vaccine
MEGA	molecular evolutionary genetic analysis
BMCMC	Bayesian Markov chain Monte Carlo
