# Supplementary material for: Genetic Diversity and Epidemic Types of Porcine Reproductive and Respiratory Syndrome (PRRS) Virus in Japan from 2018 to 2020
Source: Epidemiologia (Basel). 2022 Jun 3;3(2):285–96. doi: 10.3390/epidemiologia3020022 (PMC9620939; doi:10.3390/epidemiologia3020022)
Supplement: Supplementary file 1 [file epidemiologia-03-00022-s001.zip › epidemiologia-1695267-supplementary.pdf]

Table S1. The numbers of PRRSV nucleotide sequence isolates analyzed in a previous study (Iseki et al.) and in this study

	2007 to 2008*	2018 to 2020**	P-value
ClusterI	0	118	0.76
ClusterII	3	1177	0.042
ClusterIII	12	228	<0.01
ClusterIV	1	951	0.018
ClusterV	0	8	1
total	16	2482	NA

* Number of isolates analyzed by Iseki et.al,2011.

** Number of isolates analyzed in this study.

NA Not applicable

Pearson's Chi-square test was used to investigate the difference in the relative proportions of the isolates classified in the same cluster between the two periods.
